# Rhombencephalitis and Coxsackievirus A16

**DOI:** 10.3201/eid1510.090594

**Published:** 2009-10

**Authors:** Kazuna Goto, Masafumi Sanefuji, Koichi Kusuhara, Yorihiro Nishimura, Hiroyuki Shimizu, Ryutaro Kira, Hiroyuki Torisu, Toshiro Hara

**Affiliations:** Kyushu University, Fukuoka, Japan (K. Goto, M. Sanefuji, K. Kusuhara, R. Kira, H. Torisu, T. Hara); National Institute of Infectious Diseases, Tokyo, Japan (Y. Nishimura, H. Shimizu); 1 Current affiliation: University of Occupational and Environmental Health School of Medicine, Kitakyushu, Japan.

**Keywords:** Coxsackievirus A16, rhombencephalitis, hand, foot, and mouth disease, HFMD, letter

**To the Editor:** Hand, foot, and mouth disease (HFMD) is a common illness in children and is mainly caused by coxsackievirus A16 (CA16) and enterovirus 71 (EV71). Although its clinical course is usually uneventful and most patients experience a full recovery, serious neurologic complications, including encephalitis, can occur secondarily to HFMD caused by EV71. Such neurological complications occurred during an epidemic in Taiwan in 1998 (*1*). Encephalitis caused by EV71 is characterized by rhombencephalitis, which is a combination of brainstem encephalitis and cerebellitis. Signs and symptoms of rhombencephalitis are irritability, myoclonus, ataxia, and cranial nerve involvement (*1*). In contrast to EV71, HFMD caused by CA16 is associated with few neurologic complications with the exception of infrequent aseptic meningitis (*2*). We report a case of rhombencephalitis that developed in an infant as a complication of HFMD caused by CA16.

HFMD was diagnosed in a 23-month-old girl on the basis of high fever (>40°C, 3 d duration), stomatitis, and multiple papules on her palms, soles, and buttocks. Her illness occurred in the summer of 2007, when sentinel surveillance in the region indicated an epidemic of HFMD caused by both CA16 and EV71. She was admitted to our hospital in Fukoka, Japan, on day 4 of illness because of abnormal eye movement, irritability, and inability to stand. She had intermittent to-and-fro, horizontal oscillations of the eyes (ocular flutter). She also had truncal and limb ataxia and myoclonus in her head and limbs. Brain magnetic resonance imaging (MRI) showed T1-low and T2-high bulbopontine and cerebellar lesions around the fourth ventricle (Figure). Peripheral blood showed a mild leukocytosis (13.13 × 10^9^/L) and a C-reactive protein level within reference range (0.9 mg/L). Blood chemistry results were unremarkable. Cerebrospinal fluid (CSF) examination showed mononuclear pleocytosis (74/µL) with normal protein and glucose levels. CA16 was isolated from her stool specimen on day 4 of illness. Based on reverse transcription–PCR, CSF was negative for enterovirus RNA.

**Figure:**
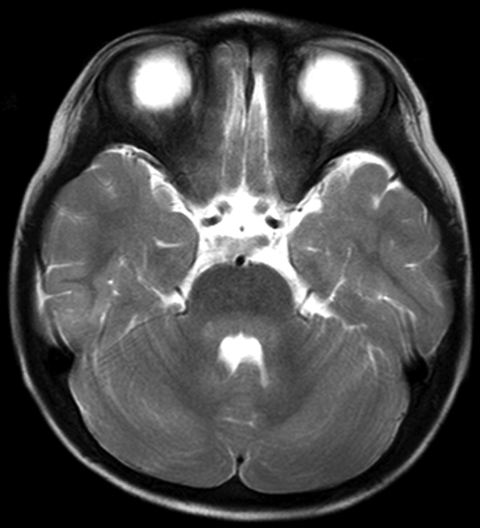
Axial T2-weighted slice of brain by magnetic resonance imaging, showing hyperintensity lesions in the pons and cerebellum around the fourth ventricle.

Without specific treatment, our patient’s fever resolved on day 5 of illness. The myoclonus, ocular flutter, and irritability subsided by day 16, when MRI findings returned to normal. Ataxia disappeared gradually ≈1 month after onset, and no neurologic sequelae occurred. Neutralizing antibody titers against CA16 and EV71 on day 21 of illness were 32 and <8, respectively. Based on the sequence analysis of the partial VP1 region (876 bp), we classified the patient’s CA16 strain phylogenetically as genetic lineage C (*3*). This lineage was identical to lineage 2 (*4*), which became the dominant circulating strain in Asia, including Japan, after the late 1990s (98.2% identical to the 1018T/VNM/05 strain isolated in Vietnam in 2005 [GenBank accession no. AM292441]) (*4*,*5*).

The patient’s symptoms of irritability, ataxia, myoclonus, and ocular flutter 3 days after the onset of typical HFMD manifestations, along with CSF mononuclear pleocytosis and the lesions around the fourth ventricle shown on MRI, led to the diagnosis of rhombencephalitis associated with HFMD. Virologic examination, including virus isolation and antibody assay, suggested that HFMD was caused by CA16 but not by EV71, although the possibility that CA16 infection was coincidental to the rhombencephalitis could not be excluded.

Although rhombencephalitis can be related to various infectious agents (*6*), HFMD complicated by this condition has been exclusively caused by EV71 (*1*,*7*,*8*). In Japan, CA16 and EV71 are consistently the 2 major causative agents of HFMD (*9*). EV71 infection is much more frequently associated with serious neurologic complications and fatalities than is CA16 (*2*). Since 1997, several HFMD outbreaks with multiple cases of severe neurologic pathologies have occurred in the Asia-Pacific region including Malaysia, Taiwan, and Western Australia (*1*,*7*,*8*). These complications were associated exclusively with EV71.

Why rhombencephalitis developed in our patient with CA16-related HFMD is unclear. One possibility is that the CA16 strain might have acquired neurovirulence by genetic recombination with EV71; phylogenetic evidence supports the possible occurrence of intertypic recombination involving EV71 and CA16 (*10*). Through phylogenetic analysis of the VP1 sequences, we classified the CA16 strain isolated from the patient’s stool phylogenetically as genetic lineage C (*3*), a lineage which was identical to lineage 2 (*4*). Genetic recombinations among enteroviruses occur mainly in noncapsid regions (*10*). We did not conduct phylogenetic analysis of the noncapsid regions of the patient’s CA16 strain because sequence data on the regions were very limited. Besides the viral factors, host factors, such as immune status and environmental factors, could confer susceptibility to neurologic complications of enteroviral infections.

Rhomobencephalitis associated with HFMD developed in this patient and was caused by CA16. Therefore, neurologic complications, including rhombencephalitis, should be considered even when CA16 is the prevalent virus causing HFMD.
